# Occurrence and Management of Acute, Subacute, and Delayed Toxicities in Patients with GEP-NETs Following Treatment with Radioligand Therapy

**DOI:** 10.3390/cancers18050742

**Published:** 2026-02-25

**Authors:** Ghassan El-Haddad, Linda Gardner, Hyun Kim, Heloisa P. Soares

**Affiliations:** 1 Moffitt Cancer Center and Research Institute, Tampa, FL 33612, USA; 2 Nuclear Medicine and Theranostics, UCLA Health, Los Angeles, CA 90095, USA; lgardner@mednet.ucla.edu; 3 Department of Radiation Oncology, Washington University, St. Louis, MO 63110, USA; hxkim@coh.org; 4 Huntsman Cancer Institute, University of Utah, Salt Lake City, UT 84132, USA; heloisa.soares@hci.utah.edu

**Keywords:** radioligand therapy, gastroenteropancreatic neuroendocrine tumors, adverse event, toxicity, management, radioactive contamination, radiation safety

## Abstract

Radioligand therapy (RLT) offers the ability to deliver radiation to specific cells based on the presence of certain targets. RLT with [^177^Lu]Lu-DOTATATE was approved in 2018 by the US Food and Drug Administration (FDA) for patients with gastroenteropancreatic neuroendocrine tumors (GEP-NETs). As RLT has become integrated into clinical practice, understanding the presence of and management strategies associated with acute, subacute, or delayed toxicities is essential to optimize patient outcomes. In this review article, we consider the risk factors for complications and any preventative strategies that may be employed. We present an overview of the toxicities that could occur during or soon after RLT infusion and discuss recommended management strategies, both from the literature and from experience in the clinical administration of RLT for patients with GEP-NETs. We highlight the importance of robust procedures for handling emergency situations and the potential for radioactive contamination from patients’ bodily fluids. Furthermore, the potential for delayed toxicities is also considered. In addition to toxicities and management, we also review the practical radiation safety precautions that are important both during and following RLT treatment.

## Simple Summary

Radioligand therapy is a type of treatment being developed for many cancer types, including gastroenteropancreatic neuroendocrine tumors (GEP-NETs). It uses specially designed drugs to deliver radiation directly to tumor cells within the body. Studies have shown that radioligand therapies can help some patients with GEP-NETs to live longer without disease progression. However, side effects have been reported in patients treated with radioligand therapies. Additionally, there is a risk of radiation exposure among people who come into contact with treated patients. In this article, we provide practical strategies to prevent and manage the side effects of radioligand therapy and to maintain radiation safety.

## 1. Introduction

Radiopharmaceuticals, including radioligand therapies (RLTs), provide a mechanism of directing radionuclides to target cells [1]. RLT agents comprise a radioisotope and a ligand capable of attaching to target molecules expressed on tumor cells [1,2]. The ability to deliver radiation directly to target cells limits the toxic effects on non-target cells [1,3]. The field of RLT is evolving with multiple agents now on the market or in development across various malignancies [3].

Therapeutic radiopharmaceuticals date back to the beginning of the 20th century and paved the way for research into radionuclides for cancer treatment [4]. However, it was the early 2000s that marked a significant shift in RLTs with the approval of [^90^Y]Y-ibritumomab tiuxetan and [^131^I]I-tositumomab for non-Hodgkin’s lymphoma. Although these treatments were later discontinued [5], they provided key learnings in respect of dosimetry, radiation safety, and logistics, leading to rapid clinical advances in recent years [4]. Driven by promising data from preclinical and clinical studies [6], methodological improvements including enhanced imaging-based patient selection and quantitative dosimetry, as well as more rigorous clinical trial designs [7], RLT is increasingly recognized as an effective and well-tolerated cancer therapy [4]. While numerous RLT trials across various cancers are ongoing or have been completed, there has been a notable interest in peptide receptor radioligand therapy (PRRT) for neuroendocrine tumors (NETs) [8,9,10]. The rising number of PRRT-specific trials, exploration of new indications, and expanding adoption of PRRT by institutions beyond large academic centers, suggest increasing numbers of patients may receive this treatment modality in the future. This trend underscores the need to examine the safety considerations specific to PRRT in patients with NETs. The first PRRT for NETs, [^177^Lu]Lu-DOTA-TATE (^177^Lu-DOTATATE), was approved by the US Food and Drug Administration (FDA) in 2018 [11] for the treatment of adults with somatostatin receptor-positive gastroenteropancreatic (GEP)-NETs in the United States [12]. This approval was extended to pediatric patients (≥12 years of age) in 2024 [13].

Well-differentiated GEP-NETs account for approximately two-thirds of all NETs [14] and have shown increasing incidence over the past few decades [15], likely owing to improved diagnostic and imaging tools leading to better and earlier detection [16]. However, many patients are still diagnosed with unresectable or metastatic disease [17]. Somatostatin analogs (SSAs) are recommended for advanced and metastatic NET treatment [18,19] to relieve symptoms and/or tumor growth control [20,21,22,23]. Treatment outcomes for these patients have further improved with the introduction of ^177^Lu-DOTATATE into the treatment landscape [24]. ^177^Lu-DOTATATE is a type of RLT used to treat advanced, well-differentiated NETs that express somatostatin receptors, particularly somatostatin receptor subtype 2 (SSTR2) [12]. It combines an SSA, DOTATATE, with the radioactive isotope lutetium-177. DOTATATE binds selectively to tumor cells that express somatostatin receptors, allowing for targeted delivery of radiation directly to tumors [11,25]. Once bound, the lutetium-177 emits beta particles that damage the tumor DNA, leading to tumor cell death [12,26].

With the growing number of available therapies for NETs, the optimal treatment sequencing strategy to maximize overall survival is yet to be established [27]. However, studies have shown that PRRT improves progression-free survival of patients with well-differentiated GEP-NETS when compared with SSAs and targeted therapy [28,29,30]. In view of the rapid adoption and increased use of PRRT, uniform guidelines concerning safety management may be lacking. Indeed, while the literature contains published studies on the safety of PRRTs in the treatment of NETs [31,32,33,34], there is a need to collate and examine all possible complications that may arise during or soon after its administration, whether these be acute, delayed, or requiring emergency care, and discuss recommendations on how these may be managed in practice in order to optimize treatment of GEP-NETs with PRRT. In addition, summarizing the guidelines and recommendations facilitate their use by clinicians who may be less experienced with this therapy. Therefore, in this narrative review, we explore common acute toxicities associated with administration of PRRT to patients with GEP-NETs and present the management strategies that are currently employed. We also discuss strategies for managing emergency situations in patients undergoing RLT.

## 2. General Guidance for the Management of Toxicities Associated with PRRT Administration

Generally, RLTs are well tolerated; however, adverse events (AEs) have been reported and should be carefully monitored in the course of treatment. Acute toxicities are best managed by a multidisciplinary care team, including nuclear medicine physicians, radiation oncologists, medical oncologists, nurses, and/or radiation safety officers [35].

**Figure 1:**
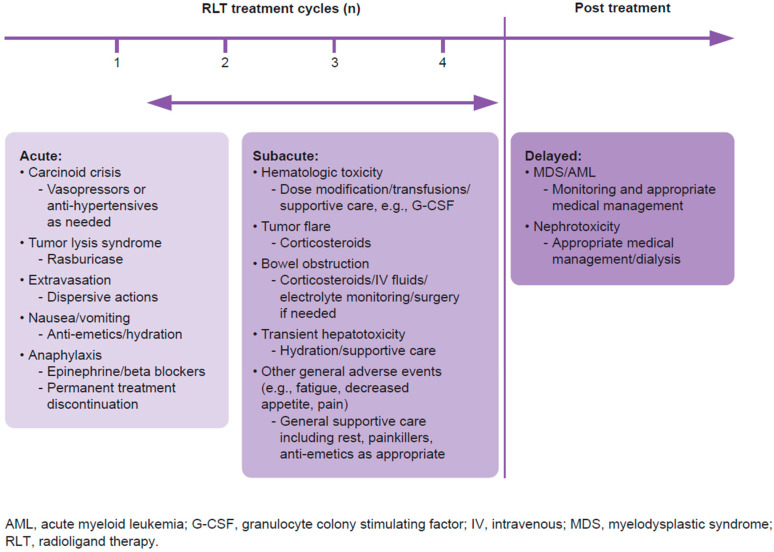
Toxicities encountered with PRRT and possible management strategies, grouped by typical occurrence timing.

PRRT dosage interruption or modification can be considered according to the time of onset and severity of AEs (e.g., clinical symptoms such as diarrhea or flushing and/or hematotoxicity [36]; Table 1). After withholding treatment due to AEs, reinitiating PRRT is recommended after a 16-week interval [12,37]. For the purposes of this review, and as illustrated in Figure 1, we have grouped toxicities into acute (arises quickly, either during or within a week after administration), subacute (arises over an intermediate timeframe, e.g., weeks or up to 3 months post treatment), and delayed (arises over a long time period, months to years after treatment has finished). Table 2 displays prevention and management strategies for some of these acute or subacute toxicities.

Guidance for the management of select co-existing medical conditions and patient concerns related to receiving RLT are explored in Supplementary Tables S1 and S2, respectively. Given the nature of this article, a detailed meta-analysis of the included studies is outside the scope of the review.

## 3. Acute Toxicities

### 3.1. Extravasation

Extravasation, where a drug being administered by infusion leaks from the vein into the surrounding tissue, may occur due to a variety of reasons from iatrogenic (e.g., poor placement of the needle) to patient-related (e.g., patient venous rupture) [60,61]. While extravasation of RLT is rare—with only isolated severe cases reported in the literature, primarily involving agents used in clinical research and not available on the commercial market—care must be taken to minimize the risk of damage to surrounding tissue [62]. In the United States, the Nuclear Regulatory Commission (NRC) is currently developing policies that may require reporting of certain radionuclide injection extravasations, particularly those with the potential to cause radiation injury—a final ruling is still pending [63]. Separately, extravasation of amino acid infusions during PRRT has been observed in clinical practice and can be severe if not promptly recognized due to the large volume of fluid administered [64].

#### Management of Extravasation

Rates of extravasation can be significantly reduced with improved injection techniques and staff training. Techniques for minimizing extravasation include using angiocatheters, peripheral intravenous (IV) lines, or peripherally inserted central catheter lines rather than butterfly needles [65]; performing meticulous IV catheter insertion [65]; carefully securing an inserted catheter [65]; and checking for patency of the IV line to ensure there are no obstructions prior to infusion [38]. In the case of PRRT, starting two peripheral IV lines—one for infusion of the amino acid solution and one for PRRT infusion—is recommended, as the high osmolarity of amino acid solutions can result in vein inflammation [35,66]. Patients should be monitored for swelling or pain, which should be acted on rapidly to increase clearance of the PRRT at the infusion site (Table 2) [38].

**Figure 2:**
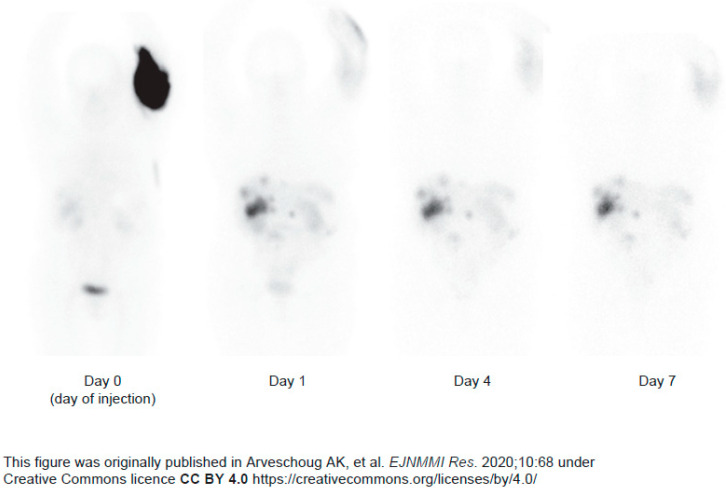
Anterior views of whole-body scintigraphies performed on Day 0, Day 1, Day 4, and Day 7. The activity initially located in the arm is redistributed to organs and tumors in the abdominal region [67].

In instances of extravasation, administration should be stopped immediately and all equipment retained in order to measure and document activity (Table 2) [39]. Light compression with bandages and heated gel pads alongside elevation is recommended to facilitate redistribution of the PRRT, which can be monitored by imaging (Figure 2) [38,39]. Surgical consultation should be obtained whenever there is concern for a severe extravasation injury, including if the patient develops severe pain; progressive swelling or pain; decreased capillary refill; changes in sensation; worsening active or passive range of motion in the elbow, wrist, or fingers; or skin ulceration or blistering [65].

Since severe extravasation injuries can develop slowly (up to hours after an extravasation), all discharged outpatients should be given clear instructions concerning where and when to seek additional medical care [65].

### 3.2. Nausea and Vomiting Related to Amino Acid Infusion

Positively charged amino acids are routinely co-infused with radiolabeled SSAs to reduce the tubular reabsorption of the PRRT, thus limiting potential renal toxicities [68]; however, they have been associated with AEs such as nausea and vomiting [69]. Studies have indicated that an arginine and lysine mix may be better tolerated with lower rates of nausea and vomiting than high osmolality amino acid mixes [40,70].

#### Management of Nausea and Vomiting Related to Amino Acid Infusion

Prevention: Premedication with antiemetics (e.g., ondansetron, fosaprepitant, or metoclopramide) may be considered for patients receiving amino acid solutions to mitigate nausea and vomiting (Table 2) [41].

Treatment: Additional antiemetics may be administered as needed (Figure 1), and decreasing the rate of amino acid infusion may also be beneficial based on clinical experience (Table 2). As delayed nausea or vomiting may occur, patients can be issued with a prescription for antiemetics should they be required post discharge.

### 3.3. Carcinoid Crisis

**Figure 3:**
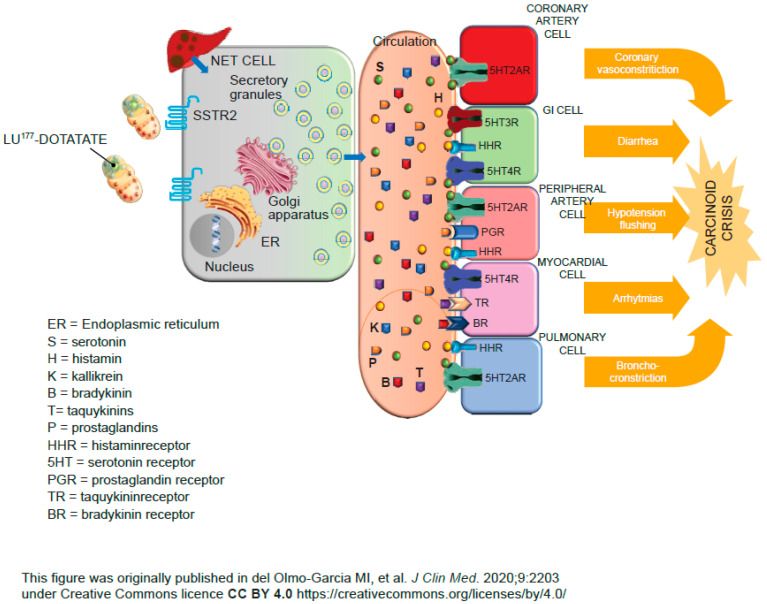
Physiopathology of carcinoid crisis during ^177^Lu-DOTATATE treatment [43].

Carcinoid crisis is a medical emergency caused by excessive release of metabolically active amines or peptides. Symptoms include flushing, hypotension or hemodynamic instability, diarrhea, bronchoconstriction, and arrhythmias (Figure 3) [43,71]. Based on the body of literature, the incidence of carcinoid crisis as a result of ^177^Lu-DOTATATE treatment is low (~1–10% of patients) and occurs most commonly with the first administration of treatment [43]. Indeed, in a joint analysis of two phase II studies of 68 patients with progressive metastatic functioning NETs and SSA-refractory syndrome, there were no reports of carcinoid crisis [72]. Patients with tumors located in the midgut, higher tumor burden, liver metastases, elevated 5-hydroxyindoleacetic acid levels, a history of carcinoid crisis, and carcinoid heart disease appear to be at increased risk of developing carcinoid crisis [43,73].

In a study of 479 patients (476 with GEP-NETs and three with metastatic pheochromocytoma) who had received ^177^Lu-DOTATATE, six patients developed severe symptoms as a result of carcinoid crisis following their first dose of treatment. All patients developing a crisis had extensive metastatic disease, with all exhibiting liver metastases and pre-existing clinically overt symptoms such as flushing and diarrhea. Crises developed during or directly after ^177^Lu-DOTATATE administration in half the patients and within 48 h of administration in the remaining patients. Patients with vasoactive intestinal polypeptide-producing pancreatic endocrine tumors or bronchial carcinoids appeared to be at higher risk of carcinoid crisis [71].

#### Management of Carcinoid Crisis

There is no clear standardized strategy to prevent carcinoid crisis. For high-risk patients, including those with carcinoid tumors, careful monitoring while receiving PRRT is recommended (Table 2) [39]. Acute carcinoid crisis management prioritizes rapid hemodynamic stabilization; vasopressors (e.g., vasopressin or phenylephrine) are first line for hypotension, while antihypertensives, such as alpha or beta blockers, address hypertension (Figure 1; Table 2) [43]. Octreotide is used adjunctively, especially early in severe cases, alongside fluids and intensive care if needed. Evidence on the role of SSAs in management of carcinoid crisis is contradictory, with some studies noting a rapid resolution of the critical status upon SSA treatment while others suggest that SSA administration had little to no effect [45]. The ideal scheme and dosing of SSAs (such as octreotide) have not been clearly established [44]. Prophylaxis with octreotide is still recommended for patients with carcinoid syndrome to prevent development of carcinoid crisis (Table 2) [44].

Following treatment, most patients who experience carcinoid crisis should be able to continue ^177^Lu-DOTATATE treatment. If required, administration activity may be reduced; however, it is important to understand that this might result in a lower tumor-absorbed radiation dose that might be less effective [71,73,74].

Patients experiencing carcinoid crisis may need transport to a hospital acute care facility, assuming that they were treated in an outpatient setting; institutional policies and procedures should be in place to deal with these situations [38]. Certain precautions should be considered ahead of treatment, including having a physician with admitting privileges present (particularly in outpatient settings) to ensure that there are no delays in the patient receiving emergency care. Being prepared and equipped to manage carcinoid crisis if it occurs, may allow for direct treatment and potentially rapid reversal of carcinoid crisis [44], thereby preventing referral to the emergency department.

### 3.4. Tumor Lysis Syndrome

Rapid destruction of tumor cells releases a large amount of intracellular content, such as potassium, phosphorus, and uric acid. This has the potential to overwhelm excretory systems, which may lead to serious complications such as renal failure, arrhythmia, or central nervous system toxicity [75]. A study that retrospectively evaluated the incidence of tumor lysis syndrome found that clinical tumor lysis syndrome developed in four of the 205 patients who had received PRRT. All four patients developed acute renal failure, and mild hyperkalemia was noted in two patients; no patients developed cardiac arrhythmia or seizures [75].

While incidence of tumor lysis syndrome appears to be low, patient monitoring for tumor lysis syndrome, particularly for patients with bulky tumors and extensive metastases [48], should be carried out when administering PRRT.

#### Management of Tumor Lysis Syndrome

Patients with tumor lysis syndrome should be treated with rasburicase (Figure 1; Table 2) at a dose of 0.2 mg/kg/day as a 30-min infusion. Intractable fluid overload, hyperkalemia, hyperuricemia, hyperphosphatemia, or hypercalcemia are indications for renal dialysis that should continue until there is adequate recovery of renal function, resolution of severe electrolyte imbalance, and recovery of urine output [47].

Identifying high-risk patients prior to treatment and administering appropriate preventative medication may lower the risk of tumor lysis syndrome [42,47]. Patients with particularly large tumors may be at higher risk of tumor lysis syndrome and careful monitoring of these patients while receiving PRRT is recommended [39].

### 3.5. Anaphylaxis

While allergic reactions are rare, type I hypersensitivity reactions and anaphylactic shock have been reported with radiopharmaceuticals [76,77].

#### Management of Anaphylaxis

As shown in Figure 1 and Table 2, anaphylaxis management primarily includes epinephrine autoinjectors; beta blockers and angiotensin-converting enzyme inhibitors can be considered based on the benefit–risk for the patient [49]. In accordance with the prescribing information for ^177^Lu-DOTATATE, patients should be monitored for hypersensitivity reactions, including anaphylaxis; ^177^Lu-DOTATATE might need to be permanently discontinued (no dose reductions) based on the severity of the reaction [12].

### 3.6. Management of Emergency Situations

Patients receiving RLT usually have advanced disease, which may increase the risk of acute toxicities and the need for unplanned emergency care [78]. In a study of 232 patients who had received PRRT infusions, 19 patients required emergency care within 7 days of PRRT infusion; two of these were a result of carcinoid crisis [78]. Although the need for emergency care was infrequent, the authors suggested that all centers administering PRRT should have robust procedures in place to effectively care for radioactive patient emergencies while keeping the treating healthcare professionals safe [78]. RLT administration must be performed in compliance with local radiation safety regulations. Training of radiation safety personnel in the safe use of unsealed radiation sources, waste management, and handling of accidental contamination is paramount [39].

For patients requiring overnight stays, typically due to complications, nursing staff need to be instructed in radiation safety precautions, given, for example, the potential for contamination from the patient’s bodily fluids, particularly urine. Ideally, radiation safety staff would prepare the patient’s bed, floor, and bathroom to minimize the risk of contamination [35]. Institutions should prepare radiation safety guidelines that include approaches to medical emergencies or patient deaths [35]. Having a standardized rapid response protocol in place, with clear delineation of roles and responsibilities, will help to ensure that patient’s health is prioritized while minimizing the risks of contamination spread and exposure to staff [79]. In the event of a patient death within days of PRRT, it is important to notify the local Radiation Safety Officer who should instruct the medical examiners and mortuary personnel on how to handle the body [35].

Emergency department (ED) providers should follow simple radiation safety steps such as standard time–distance–shielding practices, routine use of personal protective equipment, careful handling of bodily fluids, and early notification of the Radiation Safety Officer or designee [79]. These measures ensure staff safety while reinforcing that patient care should never be delayed due to radiation concerns. A checklist for key considerations in the ED is provided in Table 3.

## 4. Subacute Toxicities

### 4.1. Hematologic Toxicity from Myelosuppression

As a result of irradiation of the bone marrow and subsequent damage to hematopoietic tissue, a common adverse event of ^177^Lu-DOTATATE treatment is hematologic toxicity [29,80,81]. Despite reports of patients developing grade ≥ 3 hematologic toxicity following treatment with ^177^Lu-DOTATATE [81,82], treatment discontinuation was rarely required [82]. In the recent NETTER-1 trial (NCT01578239), in 111 patients receiving ^177^Lu-DOTATATE, lymphopenia was reported in 18% of patients, leukopenia in 10%, neutropenia in 5%, thrombocytopenia in 25%, and anemia in 14% [29]. Similarly, in a safety subanalysis of the NETTER-2 trial (NCT03972488), among 147 patients treated with ^177^Lu-DOTATATE, lymphopenia was reported in 38.1%, leukopenia in 4.1%, neutropenia in 3.4%, thrombocytopenia in 2.0%, and anemia in 1.4%; these events were transient and manageable [83]. The infection rate was similar between ^177^Lu-DOTATATE and control arms [83].

The presence of initial cytopenia prior to treatment and cumulative administered activity were associated with developing hematologic toxicities [84]. The presence of bone marrow metastases has also been associated with hematologic toxicity in some smaller retrospective studies [85,86]; however, this has not been universally seen [84]. Clinical complications of toxic bone marrow aplasia have been reported in a case study of a patient who was treated with ^177^Lu-DOTATOC in combination with capecitabine and temozolomide (CAPTEM). The treatment was initially well tolerated, but the patient was hospitalized 23 days later with neutropenic fever, a right-sided necrotizing mastitis, and fungal pneumonia. The authors concluded that the establishment of algorithms incorporating predictors of myelotoxicity would be desirable to assist selecting optimal treatment strategies [56].

#### Management of Hematologic Toxicity

For the management of hematologic toxicities, dose modification should be considered in cases of grade 3–4 hematologic toxicity [12,36] (Figure 1; Table 1). In practice, we have also considered a significant drop in platelet counts, even if lower than grade 3 in severity, as a reason to decrease the administered activity. Transfusions of red blood cells and/or the administration of granulocyte colony stimulating factors may be considered in cases of anemia or neutropenia (Figure 1; Table 2) [58,59]; however, where possible, transfusions should be avoided on the same day as PRRT administration to avoid potential fluid overload.

### 4.2. Tumor Flare

Tumor flare reactions have been noted in patients following worsening symptoms of bone or soft tissue metastasis. In a recent publication, five patients experienced flare reactions with symptoms related to metastatic sites ranging from bone pain, epigastric pain and early satiety, increased bowel dysfunction, and partial small bowel obstruction. Patients with symptomatic GEP-NET lesions prior to treatment may be at a higher risk of flare reactions [46].

#### Management of Tumor Flare

Flare reactions can be managed with a short course of corticosteroids as well as symptomatic medications (Figure 1; Table 2) [46]. Glucocorticoids can downregulate SSTR2 receptors, and therefore high doses or prolonged use should be avoided for patients prior to receiving PRRT [41]. Although there are no studies to guide the decision of steroid use, most prescribed strategies included methylprednisolone from 16 mg up to 125 mg, prednisone from 15 mg up to 20 mg, or dexamethasone from 8 mg up to 100 mg [87].

### 4.3. Bowel Obstruction

Patients with extensive mesenteric or peritoneal involvement at baseline may be at higher risk of radiation-induced bowel obstruction [38,50,51]. In a single center study of patients treated with ^177^Lu-DOTATATE, of 81 patients with mesenteric or peritoneal disease, 6% experienced at least one episode of bowel obstruction within 3 months of treatment [54]. The etiology of obstruction is likely to be related to the inflammatory response induced by radiation of the treated tumors [54]. In another retrospective study of 52 patients with small bowel tumors and mesenteric masses larger than 2 cm, four patients presented with gastrointestinal complications during ^177^Lu-DOTATATE treatment, including one patient who experienced bowel obstruction [53]. Three patients experienced bowel obstruction during long-term follow-up after treatment [53]. This potential for PRRT to initially exacerbate obstructive symptoms in patients with mesenteric masses was also noted in two recent case studies [52]. Radiation peritonitis has been noted in some patients receiving PRRT that can lead to a frozen abdomen with an irreversible intestinal blockage [50]. Caution should also be exercised when treating patients with mesenteric vessel encasement, whether arterial or venous, as acute bowel ischemia has been noted as a rare complication of PRRT in these patients [88].

#### Prevention and Management of Bowel Obstruction

Figure 1 and Table 2 outline strategies for managing bowel obstruction. Empiric treatment with a short course of corticosteroids during or immediately after RLT may reduce the possibility of obstruction occurring related to inflammation of the mass [54]. This may be of particular importance in patients who present with mesenteric masses, extensive peritoneal metastases, or omental caking [52,53,54]. However, it should be noted that data are largely from retrospective studies and a prospective randomized study would be useful in determining whether prophylaxis with corticosteroids in these patients with mesenteric masses would be beneficial in avoiding bowel obstruction [52,53,54]. In instances of complete obstruction, surgery should be considered [55]. Should surgery be required within 72 h of PRRT administration, radiation exposure should be considered and a Radiation Safety Officer informed in order to ensure correct radiation safety procedures are used.

Non-operative management of small bowel obstruction includes IV fluid resuscitation, electrolyte monitoring and supplementation, nil per os (nothing by mouth) status, nasogastric decompression, serial abdominal exams, and avoidance or minimal use of opiates (Figure 1; Table 2) [55]. Patients should be monitored regularly and surgery considered where obstruction persists for more than 3 to 5 days (Figure 1).

## 5. Delayed Toxicities, Including AEs with Long-Term Impact

### 5.1. Myelodysplastic Syndrome and Acute Myeloid Leukemia

Myelodysplastic syndrome (MDS) and acute myeloid leukemia (AML) have been linked with treatments that cause myelosuppression [89]. MDS or AML has been reported in up to 4% of patients with NETs who have received PRRT [84,90,91]. MDS was reported in one patient (0.9%) in the NETTER-1 trial and in one patient (1%) in the NETTER-2 trial [28,29]. In patients who have been re-treated with PRRT, percentages for MDS or AML remain similar to the initial treatment [92,93]. In a recent meta-analysis of 13 studies of ^177^Lu-PRRT re-treatment in 560 patients overall, pooled MDS and AML incidence was 0% (95% CI: 0, 2) [93].

A link between prior chemotherapy and development of MDS or AML following PRRT is under investigation. A recent study of 20 patients reported a high incidence (20%) of MDS and AML after PRRT in patients who had been heavily pretreated with alkylating chemotherapy [94]. However, the results of a study with such small patient numbers have been brought into question as one of its limitations [95]. More recently, data from a study of 49 patients who had received CAPTEM and PRRT revealed that 8% of patients developed MDS or AML, which is higher than the rate expected with either CAPTEM or PRRT alone [96].

There are currently no robust pre-emptive PRRT monitoring options in place [97]. Identifying biomarkers (Figure 1) and clinical factors (e.g., clonal hematopoiesis, germline mutations, age, prior cytotoxic therapies, and baseline cytopenias) may be critical in predicting and mitigating these adverse outcomes [98].

### 5.2. Nephrotoxicity

PRRT has the potential to induce nephrotoxicity as these agents are primarily cleared through glomerular filtration; therefore, reabsorption or retainment at the renal proximal tubules exposes kidneys to additional radiation [68,99]. Nephrotoxicity is a challenge to the successful outcomes of PRRT as it has been reported as a dose-limiting AE [100]. However, in patients with normal kidney function, the risk of nephrotoxicity is low. In the NETTER-1 study, no evidence of renal toxic effects was observed in 111 patients treated with ^177^Lu-DOTATATE during the 14-month follow-up [29]. In the NETTER-2 study of higher grade GEP-NETs, nephrotoxicities of any grade were reported in 9% of 147 patients treated with ^177^Lu-DOTATATE compared with 5% of 73 patients in the high-dose octreotide control arm [28]. In a study of 610 patients treated with ^177^Lu-DOTATATE at an institute in the Netherlands, only six (1%) exhibited renal failure, and the authors considered it unlikely that PRRT was the cause, as more plausible explanations were identified [101].

Long-term nephrotoxicity has been reported after exposure to commercially available PRRTs [102,103], and potential biomarkers for nephrotoxicity have been studied in the case of an investigational alpha-particle RLT [104]. Long-term data are limited; however, a recent retrospective analysis of 1281 patients with neuroendocrine neoplasms who had received a total of 4709 cycles of PRRT co-administered with renal protection found no evidence of long-term PRRT-related renal toxicity [105].

The risk factors for developing nephrotoxicity after PRRT are poor renal function, hypertension, and diabetes [100,106]. In addition to these potential comorbidities, patients may have had exposure to several cycles of chemotherapy that are known to induce nephrotoxicity, such as cisplatin [107], thus predisposing these patients to an increased risk of renal injury following PRRT [100]; amino acid co-infusion is the standard regimen for competitive inhibition of tubular reabsorption of PRRT to mitigate nephrotoxicity. Other measures to protect renal function include hydration, modifying the characteristics of the PRRT (structure of the ligand or type of radionuclide), further mechanisms to inhibit uptake of the PRRT (e.g., use of plasma expanders, renal brush border strategy), and reducing harmful effects of radiation on the kidney (e.g., by dose fractionation, administration of renal protective agents, radioprotectants, or blockade of the renin–angiotensin–aldosterone system) [68,99].

### 5.3. Hepatotoxicity

In the randomized clinical trial NETTER-1, which evaluated ^177^Lu-DOTATATE in 111 patients with advanced, progressive midgut NETs, grade 3 and 4 liver function test abnormalities were rare and did not appear to correlate with baseline tumor burden [108]. Furthermore, retrospective studies in GEP-NETs have also reported low hepatotoxicity, even in patients with very high liver tumor burden [109,110,111]. One of these retrospective studies of ^177^Lu-DOTATATE in 354 patients with GEP-NETs reported that although aminotransferase or bilirubin levels may have increased following PRRT treatment, it was found to be largely transient [111]. In contrast, a retrospective study of 93 patients with GEP-NETs and liver metastases suggested that patients exposed to PRRT had a higher risk of hepatotoxicity than those unexposed [112]. Overall, underlying or development of ascites seems to be a predictor of hepatotoxicity [110].

Radioembolization, in which radioactive beads are implanted intra-arterially to locally irradiate liver tumors, has been used to treat neuroendocrine liver metastases [113]. Concerns over potential liver failure have been raised when combining radioembolization with PRRT. For patients who have been heavily pretreated with multiple radioembolizations, especially in the case of bilobar treatment, PRRT may no longer be considered as a treatment option in later lines of therapy [114].

### 5.4. Ascites

Ascites is a syndrome characterized by excess fluid in the peritoneum [115]. There is a possibility that radioactive material from PRRT may accumulate in ascitic fluid following treatment. In a recent case study of a patient with chylous ascites, the radioactivity of the drained peritoneal fluid was analyzed [116]. The activity was found to be exceptionally low as early as 3 days after ^177^Lu-DOTATATE treatment [116]. However, it may be important to consider paracentesis earlier to avoid radiation-induced toxicity to abdominal organs. In these cases, evaluation with single-photon emission computed tomography and computed tomography should be performed, and radiation safety staff should be present to manage contamination and waste during and after paracentesis. For patients with ascites present before PRRT, physicians may consider draining the fluid prior to treatment and, in cases where a large volume is removed, using albumin infusion to prevent post-paracentesis circulatory dysfunction [117].

### 5.5. Other Common AEs

Further commonly reported AEs following PRRT include fatigue, asthenia, abdominal pain, decreased appetite, diarrhea, and musculoskeletal pain [28,29]. For each of these AEs, general supportive care strategies should be employed as needed (Figure 1). Assessment of a patient’s symptoms and their impact on function can be performed when patients return for dosimetry imaging post treatment. Moreover, a recent study reports the inclusion of patient-reported measures using the Functional Assessment of Cancer Therapy—Radionuclide Therapy (FACT-RNT) before each cycle of treatment, results of these assessments may be helpful in clinical management and decision-making with patients receiving PRRT [118,119].

The concept of dosimetry-guided (individualized) dosing to optimize RLT efficacy (tumor response) whilst minimizing risk of radiation-related toxicity has been proposed as a way of addressing potential limitations of fixed dosing regimens [120]. In a phase II study of 52 patients with SSTR-positive NETs, patient-tailored dosing of ^177^Lu-DOTATATE (reduced administered activity of 2.78–5.55 GBq per cycle) was well tolerated, with no treatment-related grade 3 or 4 AEs reported [121]. Furthermore, a separate phase II study using renal dosimetry to individualize ^177^Lu-DOTATATE in 96 patients with NETs demonstrated encouraging efficacy and a well-tolerated safety profile with grade 3–4 AEs occurring in < 10% of patients [122]. A number of other studies—including trials such as DOBATOC (NCT04917484)—are currently evaluating whether tailoring administered activity based on patient-specific dosimetry can improve clinical outcomes compared with conventional dosing schedules. While these initiatives highlight the growing interest in personalizing RLT and may ultimately help refine future practice, further high-quality evidence is needed before such approaches can be widely adopted [123]. As this field evolves, ongoing research will clarify how best to integrate personalized dosing into routine care while ensuring practical feasibility and broad patient access.

## 6. Conclusions and Future Directions

RLTs provide a means of targeting radiation to specific tumors while minimizing off-target effects [1,3]. Over recent years, interest in the use of RLT in oncologic settings has grown rapidly and PRRT has become an important part of the treatment landscape for patients with GEP-NETs, especially following approval of ^177^Lu-DOTATATE [3,12,35]. It is important to also recognize that some specific challenges remain for PRRT treatment including variable clinician awareness and challenges with referral pathways, capacity constraints, heterogeneity of disease complicating patient selection, and the need for further established long-term safety data [9,124,125]. Future research directions will need to focus on key areas around patient selection to optimize the therapeutic index of PRRTs in patients with GEP-NETs, including improved clinical, genomic, and imaging biomarkers to identify those most likely to benefit and minimize avoidable toxicity [120]. In this regard, two genomic, blood-based biomarkers have shown potential to inform individualized management by signaling real-time tumor radiosensitivity and early disease trajectory; in an interim analysis, PRRT prediction quotient (PPQ) was a highly accurate (96%) treatment-independent predictor of ^177^Lu-DOTATATE response, and NETest changes showed 90% accuracy in monitoring of treatment response [126]. Furthermore, in addition to the development of novel combination treatment approaches and multitargeted radiopharmaceuticals, technological advances, particularly the potential opportunities afforded by artificial intelligence-driven models (e.g., in patient selection, treatment planning, dosimetry, and response assessment) [127], will play a central role in shaping the future of PRRT.

In this review we have discussed how, although relatively infrequent, some acute toxicities may occur during or shortly after PRRT administration [41,42]. It is essential that treating healthcare professionals are aware of early signs of toxicity and that robust procedures are in place for their management [35,128]. These procedures should also address the management of emergency situations and ensure collaboration with those authorized to handle potential radioactive contamination [78].

## Figures and Tables

**Table 1:** Dose modification guidelines for ^177^Lu-DOTATATE [12].

Toxicity	Severity *	Dose Modification Steps	
		Step 1	Step 2
Thrombocytopenia	First occurrence grade 2–4	Withhold dose until complete or partial resolution	If resolution: Resume ^177^Lu-DOTATATE at 50% dose. If reduced dose does not result in grade 2–4 thrombocytopenia, administer ^177^Lu-DOTATATE at full dose as next dose. If no resolution: Permanently discontinue ^177^Lu-DOTATATE for grade ≥ 2 thrombocytopenia requiring a dosing interval > 16 weeks.
	Recurrent grade 2–4	Permanently discontinue ^177^Lu-DOTATATE	
Anemia and neutropenia	First occurrence grade 3/4	Withhold dose until complete or partial resolution	If resolution: Resume ^177^Lu-DOTATATE at 50% dose. If reduced dose does not result in grade 3/4 anemia or neutropenia, administer ^177^Lu-DOTATATE at full dose as next dose. If no resolution: Permanently discontinue ^177^Lu-DOTATATE for grade 3/4 anemia or neutropenia requiring a dosing interval > 16 weeks.
	Recurrent grade 3/4	Permanently discontinue ^177^Lu-DOTATATE	
Renal toxicity	First occurrence of: Creatinine clearance < 40 mL/min calculated using Cockcroft–Gault formula with actual body weight, or 40% increase from baseline serum creatinine, or 40% decrease from baseline creatinine clearance; calculated using Cockcroft–Gault formula with actual body weight	Withhold dose until resolution or return to baseline	If resolution: Resume ^177^Lu-DOTATATE at 50% dose. If reduced dose does not result in renal toxicity, administer ^177^Lu-DOTATATE at full dose as next dose. If no resolution: Permanently discontinue ^177^Lu-DOTATATE for renal toxicity requiring a dosing interval > 16 weeks.
	Recurrent renal toxicity	Permanently discontinue ^177^Lu-DOTATATE	
Hepatotoxicity	First occurrence of: Bilirubinemia > 3 times the ULN (grade 3/4), or Serum albumin < 30 g/L with INR > 1.5	Withhold dose until resolution or return to baseline	If resolution: Resume ^177^Lu-DOTATATE at 50% dose. If reduced dose does not result in hepatotoxicity, administer ^177^Lu-DOTATATE at full dose as next dose. If no resolution: Permanently discontinue ^177^Lu-DOTATATE for hepatotoxicity requiring a dosing interval > 16 weeks.
	Recurrent hepatotoxicity	Permanently discontinue ^177^Lu-DOTATATE	
Hypersensitivity reactions (including allergic reaction or anaphylaxis)	First occurrence of grade 3/4	Permanently discontinue ^177^Lu-DOTATATE	
Any other adverse reactions ^†^	First occurrence of grade 3/4	Withhold dose until complete or partial resolution	If resolution: Resume ^177^Lu-DOTATATE at 50% dose. If reduced dose does not result in grade 3/4 toxicity, administer ^177^Lu-DOTATATE at full dose as next dose. If no resolution: Permanently discontinue ^177^Lu-DOTATATE for grade 3/4 toxicity requiring a dosing interval > 16 weeks.
	Recurrent grade 3/4	Permanently discontinue ^177^Lu-DOTATATE	

* Grading of severity is defined in the most current Common Terminology Criteria for Adverse Events (CTCAE). ^†^ No dose modification required for hematologic toxicities grade 3 or grade 4 solely due to lymphopenia. ^177^Lu-DOTATATE, [^177^Lu]Lu-DOTA-TATE; INR, international normalized ratio; ULN, upper limit of normal.

**Table 2:** Prevention and management strategies for common acute toxicities associated with PRRT administration.

Toxicity	Prevention Strategies	Management Strategies
Extravasation	Check for patency of the IV line to ensure no obstructions prior to infusion [38] Monitor the patient for swelling or pain, which should be acted on rapidly to increase clearance of the PRRT at the infusion site [38]	Stop administration immediately [39] Retain equipment to measure and document activity [38,39] Apply light compression bandages with heated gel pads to facilitate redistribution of the PRRT [38] Monitor PRRT by imaging [38,39]
Nausea and vomiting	Use a lysine and arginine mix rather than a high osmolality amino acid mix [40] Premedication with antiemetics (e.g., ondansetron, fosaprepitant, or metoclopramide) is also recommended for patients receiving amino acid solutions to mitigate nausea and vomiting [41]	Provide hydration and/or electrolytes as needed [42] Provide additional antiemetics as required Consider reducing the infusion rate of amino acids
Carcinoid crisis	Identify high-risk patients prior to treatment and administer appropriate preventative medication [42,43] Prophylaxis with octreotide may be considered in patients with carcinoid syndrome [44] Patients with carcinoid tumors or particularly large tumors may be at higher risk of carcinoid crisis and tumor debulking and/or careful monitoring of these patients while receiving PRRT is recommended [39,43]	Consider use of vasopressors (e.g., vasopressin or phenylephrine) in carcinoid crisis-related hypotension [45] Consider antihypertensives, such as alpha or beta blockers in carcinoid crisis-related hypertension [43]
Flare reaction		Administer a short course of corticosteroids after treatment (e.g., methylprednisolone dose pack or prednisone 40 mg daily) as well as symptomatic medications [46]
Tumor lysis syndrome	Identify high-risk patients prior to treatment and administer appropriate preventative medication [42,47] Patients with bulky tumors and extensive metastases require careful monitoring [48]	Infusion of rasburicase should be considered in patients without contraindications [47] Renal dialysis may be required in patients with intractable fluid overload, hyperkalemia, hyperuricemia, hyperphosphatemia, or hypercalcemia [47]
Anaphylaxis		Epinephrine autoinjectors; beta blockers and angiotensin-converting enzyme inhibitors can be considered based on the benefit–risk for the patient [49] Treatment must be permanently discontinued (no dose reductions) based on the severity of the reaction
Bowel obstruction	Careful monitoring of patients with extensive mesenteric or peritoneal involvement at baseline [38,50,51] In patients with mesenteric masses, a short course of corticosteroids prior to treatment may be considered [52,53,54]	Treatment with a short course of corticosteroids after PRRT may reduce obstruction that is related to inflammation [54] In instances of complete obstruction, surgery should be considered [55] Non-operative management of small bowel obstruction includes IV fluid resuscitation, electrolyte monitoring and/or supplementation, NPO status, nasogastric decompression, serial abdominal exams, and avoidance or minimal use of opiates [55] Patients should be monitored regularly and surgery considered where obstruction persists for more than 3–5 days [55]
Hematologic toxicity	Careful monitoring of patients with extensive bone metastases or other predictors of myelotoxicity [56]	Dose adaptation may be applied to allow for resolution of acute toxicity [36] In patients with diffuse bone marrow infiltration of NET, peripheral blood stem cell collection may have potential as a method to overcome dose-limiting bone marrow toxicity [57] Red blood cell transfusions are required for around 15% of anemic patients with cancer [58] Neutropenia can be minimized using granulocyte colony-stimulating factors in high-risk patients [59]

IV, intravenous; NET, neuroendocrine tumor; NPO, nil per os (nothing by mouth); PRRT, peptide receptor radionuclide therapy.

**Table 3:** Example of ED checklist for care of patients presenting within 7 days of radioligand therapy (^177^Lu-PSMA, ^177^Lu-DOTATATE, and similar therapies).

Item	Actions/Comments
1. Reassure and prepare ED staff	Residual radiation levels are low with routine precautions Offer electronic personal dosimeters if available for real-time exposure reassurance Contextualize exposure (e.g.,a cross-country flight ≈ 3.5 mrem) Maintain a calm, structured workflow
2. Do NOT delay medical care	All diagnostics and urgent interventions are permitted • CT, MRI, US • Labs and IV access • Resuscitation and emergency procedures Clinical urgency always takes priority
3. Follow Time–Distance–Shielding	Time: Limit unnecessary close contact Distance: Maintain > 1 m when not performing care Shielding: Existing ED shielding is sufficient
4. Notify RSO immediately	Contact RSO upon learning patient received RLT within 7 days EMR tip: Enable automatic RSO alerts when an RLT-treated patient registers or is admitted RSO responsibilities: ◦ Assess contamination risk ◦ Advise on signage (e.g., *Caution: Radioactive Materials*) ◦ Guide waste handling ◦ Support staff ◦ Perform room survey on discharge before returning room to routine clinical use
5. PPE and room preparation	Standard PPE: gloves, gown, eye protection Shoe covers for anyone entering the room If feasible: Place tarp or waterproof barrier on floor near bed/toilet Help prevent inadvertent spread from spills or splashes
6. Handling of bodily fluids	Highest residual activity: urine, followed by stool, emesis, wound drainage Use strict contact precautions Handle urinals with extra care to avoid spills—these can trigger regulatory contamination issues Bag contaminated materials; store for RSO guidance Do not dispose in general waste unless cleared by RSO
7. Room selection and isolation	Strongly recommend a private room Avoid shared rooms to prevent cross-contamination Use designated or single-patient bathroom, if possible If shared bathroom used → RSO must survey before reopening
8. Limit staff rotation	Designate one primary nurse and one physician whenever feasible Reduces cumulative exposures and contamination opportunities
9. Document key treatment details	Record: • Date of RLT • Radiopharmaceutical name • Administered activity (if available)
10. “Just in Time” training resources	Provide ED staff with a laminated quick reference card including: ◦ This checklist ◦ RSO and NM contact numbers ◦ Spill procedures ◦ PPE reminders ◦ Room release steps Keep copies at triage, charge desk, and staff work stations

CT, computed tomography; ED, emergency department; EMR, electronic medical record; IV, intravenous; MRI, magnetic resonance imaging; NM, nuclear medicine; PPE, personal protective equipment; RLT, radioligand therapy; RSO, radiation safety officer; US, ultrasound.

## Data Availability

Not applicable.

## Supplementary Materials

The following supporting information can be downloaded at: https://www.mdpi.com/article/10.3390/cancers18050742/s1, Table S1: Considerations for the management of select co-existing medical conditions [35,129,130,131,132]; Table S2: Patient concerns related to receiving RLT [133,134,135,136]; Table S3: Example institutional guidance for patients following ^177^Lu-DOTATATE treatment; Figure S1: Examples of wallet cards issued to patients following each ^177^Lu injection [137].
