# Supplementary material for: Occurrence and Management of Acute, Subacute, and Delayed Toxicities in Patients with GEP-NETs Following Treatment with Radioligand Therapy
Source: Cancers (Basel). 2026 Feb 25;18(5):742. doi: 10.3390/cancers18050742 (PMC12984351; doi:10.3390/cancers18050742)
Supplement: Supplementary file 1 [file cancers-18-00742-s001.zip › cancers-4024021-supplementary.pdf]

Supplementary Appendix to:

Occurrence and Management of Acute, Subacute, and Delayed Toxicities in Patients with GEP-NETs Following Treatment with Radioligand Therapy

Ghassan El-Haddad, Linda Gardner, Hyun Kim and Heloisa P. Soares

Supplementary Table S1. Considerations for the management of select co-existing medical conditions.

Condition	Considerations
Patients who require use of colostomy bag and/or urinary catheter or patients with incontinence	• Following administration of ^{177}Lu-DOTATATE, radiation is primarily excreted through the kidneys via urine and, to a lesser extent, stool [35]<br>• Healthcare professionals caring for these patients should be aware of the potential for radioactive contamination from bodily fluids and the need for wearing gloves and gowns when handling waste
Patients with oliguria/anuria and requiring hemodialysis	• Patients are at higher risk for delayed elimination of the radiation and, hence, possible additional radiation exposure<br>• Despite no standardized recommendations for patients on hemodialysis, different institutions have reported successful treatment of such patients. A recent literature review of publications, NANETS or ENETS recommendations, and ^{177}Lu pharmacokinetics found seven relevant publications with a total of 15 patients. Patients received dose-adjusted fractions of PRRT with hemodialysis generally occurring within 24 hours. No immediate or long-term serious AEs were attributed to the RLT; however, it was noted that data were limited [129]<br>• Establishing institutional workflows is suggested in the absence of strong data
Patients of childbearing potential	• Guidelines on the use of PRRT in patients of childbearing potential recommend administration only after verification of a recent (within 1–5 days) negative pregnancy test, and advise that effective contraceptive methods be used throughout the treatment period and for 4–6 months after the last cycle of PRRT [35,130]<br>• For those patients who are planning to conceive children in the future, considering the possible temporary impairment of fertility, patients should consider using cryopreservation of sperm and potentially oocytes before undergoing PRRT; fertility specialist referral should be discussed [35,130-132]

AE, adverse event; PRRT, peptide receptor radioligand therapy; RLT, radioligand therapy.

Supplementary Table S2. Patient concerns related to receiving RLT.

Concern	Recommendation
Exposure of close contacts to radiation	• See **Supplementary Table S3**<br>• Institutions should work with their radiation safety office to follow local guidelines. In general, guidance relates to contact with other people, disposal of waste, and maintaining good hygiene standards that limit impact on others [133]<br>• The majority of radiation from ^{177}Lu-DOTATATE is limited to 2 mm, and does not penetrate beyond the abdominal or chest wall [134]
Exposure of pets to radiation	• As the levels of radiation are low and most pet life spans are shorter than humans, it should be safe for pets to be handled; however, if patients prefer to use caution, they can limit contact with pets in the first couple of days following treatment [135]
Travel	• Due to the low levels of radiation emitted by patients following treatment, there is a chance that this will be detected by sensitive radiation detectors at US ports of entry (including airports and border crossings) [136]<br>• Patients should be provided with a card that details the treatment they have received and states that radioactivity can be detected for several weeks following therapy (see example **Supplementary Figure S1**). The card should also include the institution where the treatment has been given and provide contact details in case further information is needed [136]<br>• Some institutions provide an after-visit summary (AVS) document available electronically via a health portal and containing pertinent information that the patient may use for presentation<br>• Licensed practitioners with appropriate authorization—commonly referred to as “Authorized Users” in the USA—along with Radiation Safety personnel, are responsible for ensuring and documenting that patients meet regulatory release criteria following radioligand therapy<br>• In the USA, each institution may follow the guidance of the US Nuclear Regulatory Commission (NRC) according to Agreement State regulations. Similar roles exist globally, with specific requirements varying by country and local regulatory authority<br>• Healthcare professionals will need to explain to patients that they may be delayed while travelling and that they should provide authorities with the card to explain the situation [136]

Supplementary Table S3. Example institutional guidance for patients following ^{177}Lu-DOTATATE treatment.*

Group	Avoid close contact	Separate sleeping	Notes
Infants (<2 years)	10–14 days	10–14 days	No hugging, holding, or lap-sitting; Limited proximity (>1 m, <2 hrs/day)
Young children (2–12 years)	5–7 days	5–7 days	Limited proximity (>1 m, <2 hrs/day)
Adolescents (12–18 years)	2–3 days	2–3 days	General distance (>1 m, <6 hrs/day)
Pregnant women	7–14 days	7–14 days	Limited proximity (>1 m, <2 hrs/day)
Adults	1–3 days	3–7 days	Separate bed recommended

*Please also consult with your local radiation safety office. The calculation (and patient-specific instructions) may show lower/higher times, depending on the patient-specific answers.

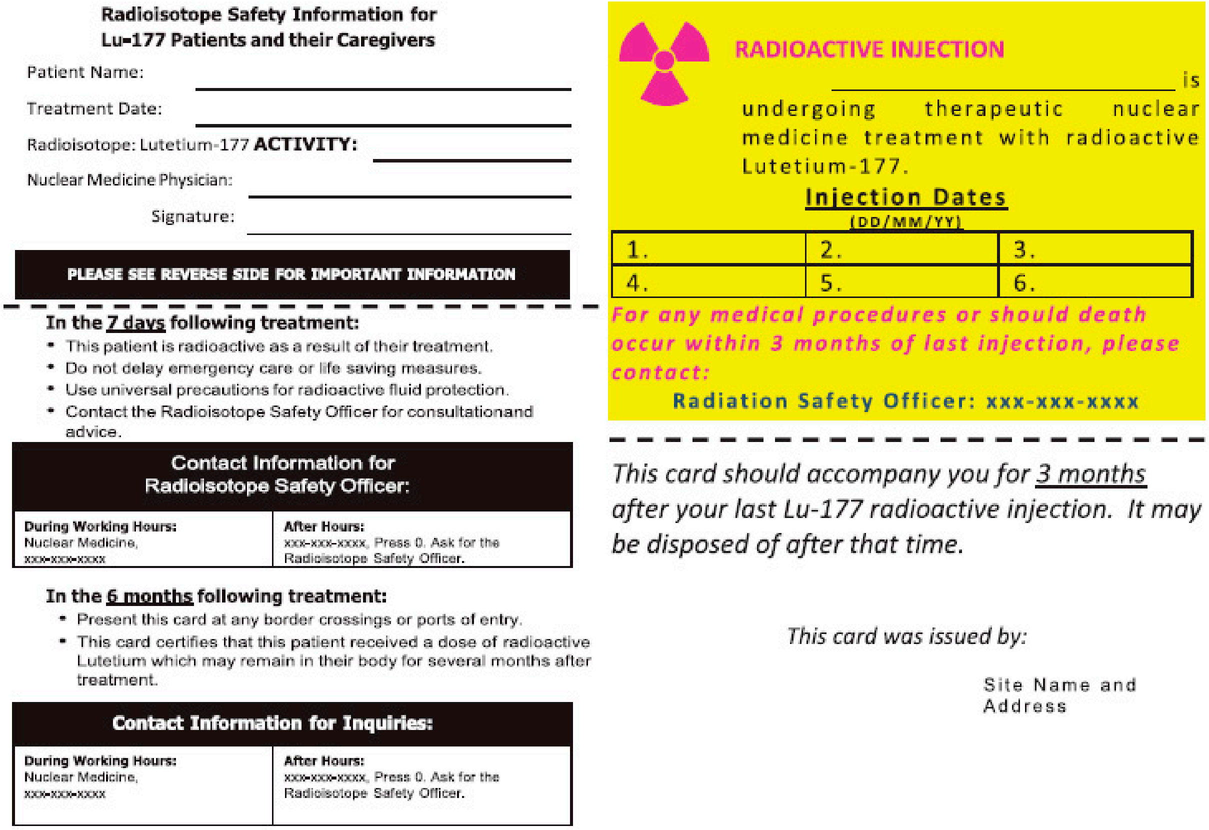

Radioisotope Safety Information for Lu-177 Patients and their Caregivers

Patient Name: ______

Treatment Date: ______

Radioisotope: Lutetium-177 **ACTIVITY:** ______

Nuclear Medicine Physician: ______

Signature: ______

PLEASE SEE REVERSE SIDE FOR IMPORTANT INFORMATION

In the 7 days following treatment:

- This patient is radioactive as a result of their treatment.
- Do not delay emergency care or life saving measures.
- Use universal precautions for radioactive fluid protection.
- Contact the Radioisotope Safety Officer for consultationand advice.

Contact Information for Radioisotope Safety Officer:	
**During Working Hours:** Nuclear Medicine, xxx-xxx-xxxx	**After Hours:** xxx-xxx-xxxx, Press 0. Ask for the Radioisotope Safety Officer.

In the 6 months following treatment:

- Present this card at any border crossings or ports of entry.
- This card certifies that this patient received a dose of radioactive Lutetium which may remain in their body for several months after treatment.

Contact Information for Inquiries:	
**During Working Hours:** Nuclear Medicine, xxx-xxx-xxxx	**After Hours:** xxx-xxx-xxxx, Press 0. Ask for the Radioisotope Safety Officer.

RADIOACTIVE INJECTION

______ is undergoing therapeutic nuclear medicine treatment with radioactive Lutetium-177.

Injection Dates
(DD/MM/YY)

1.	2.	3.
4.	5.	6.

For any medical procedures or should death occur within 3 months of last injection, please contact:

Radiation Safety Officer: xxx-xxx-xxxx

This card should accompany you for 3 months after your last Lu-177 radioactive injection. It may be disposed of after that time.

This card was issued by:

Site Name and Address

This information was originally published in Cappon DJ, et al. *Health Physics*. 2023;124:139-146
under Creative Commons licence **CC BY-NC-ND 4.0** https://creativecommons.org/licenses/by-nc-nd/4.0/

Supplementary Figure S1. Examples of wallet cards issued to patients following each ^{177}Lu injection [137].
